# Overview of processed excipients in ocular drug delivery: Opportunities so far and bottlenecks

**DOI:** 10.1016/j.heliyon.2023.e23810

**Published:** 2023-12-23

**Authors:** Sumel Ashique, Neeraj Mishra, Sourav Mohanto, B.H. Jaswanth Gowda, Shubneesh Kumar, Amisha S. Raikar, Priya Masand, Ashish Garg, Priyanka Goswami, Ivan Kahwa

**Affiliations:** a Department of Pharmaceutical Sciences, Bengal College of Pharmaceutical Sciences & Research, Durgapur 713212, West Bengal, India; b Amity Institute of Pharmacy, Amity University Madhya Pradesh, Gwalior, 474005, India; c Department of Pharmaceutics, Yenepoya Pharmacy College & Research Centre, Yenepoya (Deemed to Be University), Mangalore, 575018, India; d School of Pharmacy, Queen's University Belfast, Medical Biology Centre, Belfast BT9 7BL, UK; e Department of Pharmaceutics, Bharat Institute of Technology, School of Pharmacy, Meerut 250103, UP, India; f Department of Pharmaceutics, PES Rajaram and Tarabai Bandekar College of Pharmacy, Ponda, Goa 403401, India; g Department of Pharmaceutical Technology, Meerut Institute of Engineering & Technology, (MIET), NH-58, Delhi-Roorkee Highway, Meerut, Uttar Pradesh 250005, India; h Department of Pharmaceutics, Guru Ramdas Khalsa Institute of Science and Technology (Pharmacy), Jabalpur, Madhya Pradesh, India; i Department of Pharmacognosy, Saraswati Institute of Pharmaceutical Sciences, Gandhinagar 382355, Gujarat, India; j Department of Pharmacy, Faculty of Medicine, Mbarara University of Science and Technology, P.O Box 1410, Mbarara, Uganda; k Maharashtra Educational Society's H.K. College of Pharmacy, Mumbai: 400102.India; l Pharm-Bio Technology and Traditional Medicine Centre, Mbarara University of Science and Technology, P. O Box 1410, Mbarara, Uganda

**Keywords:** Eye, Ocular, Drug delivery, Excipient, Formulation, Polymers, Nanoparticles

## Abstract

Ocular drug delivery presents a unique set of challenges owing to the complex anatomy and physiology of the eye. Processed excipients have emerged as crucial components in overcoming these challenges and improving the efficacy and safety of ocular drug delivery systems. This comprehensive overview examines the opportunities that processed excipients offer in enhancing drug delivery to the eye. By analyzing the current landscape, this review highlights the successful applications of processed excipients, such as micro- and nano-formulations, sustained-release systems, and targeted delivery strategies. Furthermore, this article delves into the bottlenecks that have impeded the widespread adoption of these excipients, including formulation stability, biocompatibility, regulatory constraints, and cost-effectiveness. Through a critical evaluation of existing research and industry practices, this review aims to provide insights into the potential avenues for innovation and development in ocular drug delivery, with a focus on addressing the existing challenges associated with processed excipients. This synthesis contributes to a deeper understanding of the promising role of processed excipients in improving ocular drug delivery systems and encourages further research and development in this rapidly evolving field.

## 1 Introduction

**Fig. 1:**
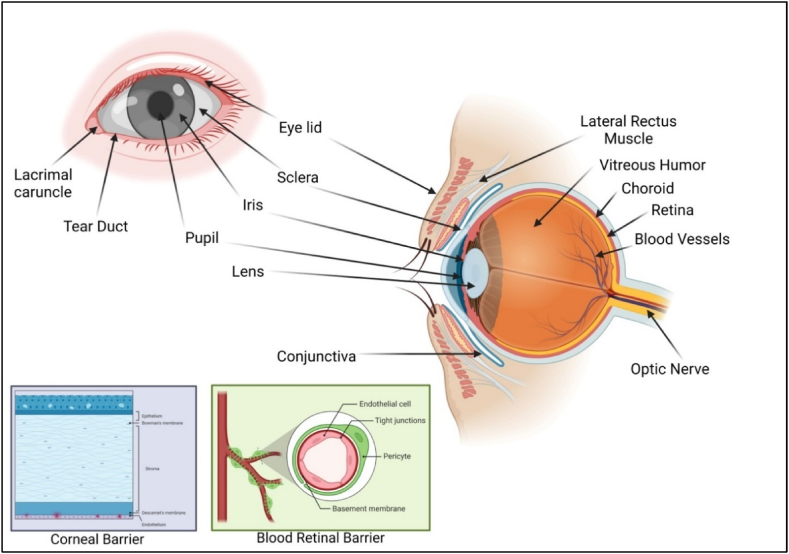
Anatomy of the human eye and its particular ocular barriers (Created with BioRender.com). The eye is divided into three layers, each of which has a barrier: (a) the exterior protecting coat of the eye, which comprises the cornea and the sclera. (b) A vascular middle layer made up of the iris, ciliary body, and choroid, and (c) an inner, neural coat made up of the retina. The cornea is a transparent trilaminar sandwich that protects the inner structures of the eye with a hydrophobic multilayered epithelium with tight junctions that connect the apical cells, a hydrophilic stroma, and a hydrophobic endothelium along with its specialized basement membrane, Descemet's membrane. The blood-aqueous barrier (BAB) and the blood-retinal barrier (BRB) are two barriers that limit the entrance of pathogenic pathogens and poisons from systemic circulation. The BAB is made up of the ciliary body's non-pigmented epithelium (NPE) and the ideal blood vessels' non-fenestrated endothelium (PE), whereas the BRB is made up of tight junctions amongst cells of the retinal pigment epithelium (RPE) and retinal vascular endothelium.

Processed excipients are essential components of pharmaceutical and medical products. They serve various functions, such as ensuring stability, improving drug delivery, and enhancing patient compliance. Excipients are inactive substances that are combined with active pharmaceutical ingredients (APIs) to create a final dosage form. Processed excipients are excipients that undergo specific treatments and processing to meet the requirements of a particular drug formulation. They can be natural or synthetic materials and are added to pharmaceutical formulations for their various functional properties. Advances in pharmaceutical technology have led to the development of novel processed excipients. These include nanoparticles, liposomes, and other specialized carriers designed to enhance drug delivery and therapeutic outcomes. The ocular system converts visual information into electrical signals, making it one of the most remarkable sensory systems in the human body [1]. Since the eye is a highly isolated organ with formidable protection, it is a challenging target for the delivery of drugs. The blood-retina barricade and the blood-aqueous humor barricade, which the ocular vascular barrier system severely limits systemic ophthalmic drug delivery. As a result, even though the cornea and conjunctiva constitute significant epithelial barriers, ocular drugs for treating the conjunctiva, cornea, and anterior chamber are given topically [1], further illustrating the basic anatomy of the eye in Fig. 1. Ophthalmic drug therapies were limited to topical treatments, injections around or inside the eye, or systemic delivery. Although ophthalmic drops are the most accessible way to deliver ocular remedies, achieving an adequate level of therapeutic substance in the targeted region for an extended period is challenging [2]. Conventional medicines are rapidly removed from ocular surfaces by baseline and reflexive lachrymation, blinking, and drainage, even before the medication contacts the optic nerve or tissues in adequate concentrations. As a result, the primary limitations in ocular medication delivery via local application include low mucosal tissue permeability and limited mucosal residence time [3]. Due to the demand for advancements in drug conveyance strategies for ocular assertions, there is a mandate to develop novel and enhanced techniques. Among the different drug delivery techniques that improve ocular bioavailability are those that provide a more extended residence period on the cornea and conjunctiva [2]. One way of achieving this is by adding new additions to existing works of art or inventing novel medication delivery systems. Drugs have a better chance of operating locally on those membranes or penetrating deeper ocular tissues because of the extended drug residence time, which allows them to achieve their target effectively. The first effective attempts at broadening the use of ocular therapies were made in the 1980s. The studies resulted in the discovery of mucoadhesive polymers that can adhere to the eye's surface, increasing the viscosity of eye drops and slowing their drainage rate [3]. The conventional mucoadhesive polymers were restricted to forming weak reversible connections with the mucus layer covering ocular surfaces during its initial phase. A new class of mucoadhesive polymers emerged due to later developments, which can form covalent bonds with mucus glycoproteins, predominantly through disulfide linkages [4].

Nanomedicine acquired significant attention in the late 1990s, prompting the development of innovative nanocarrier formulations [5]. Their small dimensions facilitate effective passage through protective layers in the eye, thereby improving the amount of drug that can be absorbed and reducing the need for frequent dosing. Through the containment of medications in tiny carriers, they safeguard sensitive eye tissues from harm, decreasing harmful reactions and optimizing the effectiveness of treatment [6]. Furthermore, their ability to circumvent the barrier between the bloodstream and the eye enables the direct administration of treatments to the specific location in need, presenting a hopeful resolution for complex eye ailments [7]. Various empirical investigations have shown that high-dosage forms such as contact lenses and eye inserts can adhere to the eye for long periods, ranging from hours to days. This insight has led to the development of multiple drug delivery systems that aim to prolong medication retention in the precorneal region. These systems have effectively enhanced bioavailability by extending the drug contact time with the eye after topical administration. As a result, many commercially available medicines have become possible through these systems [4]. Ocular drug delivery presents unique challenges due to the complex anatomy and physiology of the eye. To improve drug delivery to the eye, various processed excipients and drug delivery systems have been developed. These excipients can enhance drug solubility, improve bioavailability, extend drug retention time, and provide controlled release. Processed excipients and drug delivery systems are designed to address specific challenges in ocular drug delivery, such as limited drug absorption, rapid clearance, and the need for prolonged drug release. The choice of excipient or delivery system depends on the drug's characteristics and the desired therapeutic outcome. Additionally, regulatory approval and safety considerations are crucial when developing ocular drug delivery systems.

In the past few years, there has been extensive utilization of processed excipients in the advancement of sophisticated techniques for administering medications to the eyes, aimed at addressing a range of ocular ailments. A simple search on “PubMed” for the keywords “Excipients” and “Ocular” using “AND” as a Boolean operator resulted in 286 articles from 2010 to 2022. Furthermore, the keywords “Nanomedicine” and “Ocular” using “AND” as a Boolean operator resulted in 437 articles from 2010 to 2022. This indicates that there are ample opportunities for further advancements in creating efficient methods for delivering drugs to the eyes, employing a diverse array of processed excipients.

## 2 Effects of physiological factors on ocular drug delivery approaches

The designing and development of drug delivery systems targeting ocular tissues poses a significant challenge for scientists. The eye is alienated into frontal and later chambers, and each layer of ocular tissue presents structural variations that hinder drug absorption regardless of the administration route (i.e., topical, systemic, periocular) [8]. Due to various inherent barriers specific to the optic system anatomy and physiology, ophthalmic drug delivery offers a unique encumbrance to systemic drug delivery [9]. These barriers present significant hurdles for drug delivery scientists. Depending on the route of administration (e.g., local, enteral, parenteral, etc.), as shown in Table 1, specific biological barriers exist, which serve to shelter the eye from conceivably detrimental rudiments [10]. Overcoming these barriers requires careful consideration and innovative approaches in sending remedies to the eye [10]. The treatments for anterior eye conditions usually involve the use of topical administration, which is primarily accomplished through ophthalmic drops. Several factors could improve the efficacy of locally applied medications. Precorneal factors, i.e., tear film dynamics, blinking, tear drainage, and induced lacrimation, can significantly impact the bio-readiness of topically directed formulations [11]. The tear crust acts as a protective barrier and quickly removes administered solutions due to its high turnover rate. Due to limited contact time with absorptive membranes, the amount of the applied dose that reaches intraocular tissues is relatively low [8,12].

**Table 1:** Overview of ocular delivery methods: Routes, advantages, and challenges.

Route of Administration	Characteristics/Importance	Drawbacks/Limitations	Claims in Ocular Conveyance	References
Topical	• Convenient than non-invasive. • Easy self-direction.	• Limited drug penetration and bioavailability. • Rapid drug clearance from the ocular surface. • Low patient compliance.	Conjunctivitis, dry eye, glaucoma, allergic eye disorders	[13,14]
Subconjunctival Injection	• Prolonged drug release. • High drug concentrations at the target site.	• Invasive administration. • Risk of infection and tissue damage.	Ocular inflammation, infection, macular edema	[15,16]
Intravitreal Shot	• Direct sending to the vitreous. • Long duration of drug action.	• Risk of endophthalmitis then retinal detachment. • Frequent injections are required.	Age-related macular degeneration, diabetic retinopathy	[[17], [18], [19]]
Trans corneal permeation	• Non-invasive and easy administration.	• Limited drug permeation across the cornea. • Need for penetration enhancers or advanced techniques.	Corneal infections, anterior uveitis	[20,21]
Implantable devices	• Sustained drug release. • Reduced frequency of administrations.	• Invasive implantation procedure. • Potential complications associated with the implant.	Glaucoma, retinal diseases	[13,22,23]
Intracameral	• Results in elevated drug concentrations within the anterior chamber. • Eliminates the need for the application of topical drops. • Minimizes corneal then systemic adversative things associated with the use of topical steroids.	• Toxic frontal sector ailment. • Toxic endothelial cell demolition ailment.	Anesthesia, preclusion of endophthalmitis, irritation and pupil distention	[24]

The cornea, conjunctiva, and sclera, which form the ocular surface, present additional barriers to drug permeation. The cornea entails different coats, including the epithelium, stroma, and endothelium, separately posing encounters for drug penetration due to their specific properties and structures [12]. The attendance of lipoidal characteristics and skintight junctional complexes in the corneal surface layer poses a barricade to the pervasion of aquatic-solvable remedies. The stroma's well-hydrated structure presents a limitation for non-polar drugs. The endothelium controls the movement of fluids between the stroma and the aqueous humor. Furthermore, the blood vessels and tight junctions in the conjunctiva cause medicines to be drained into the bloodstream, which reduces their effectiveness in treating eye conditions [25].

The sclera, contiguous with the cornea, has permeability comparable to the corneal stroma. During ocular drug delivery through systemic administration (via parenteral routes), drugs face the challenge of crossing intraocular barriers [1]. The blood-eye barricade is assembled by the tight connections between the cells in the endothelium of the iris/ciliary venules and the non-pigmented ciliary epithelium. These connections act as a protective barrier that prevents solutes, including the aqueous humor, from entering the intraocular space [26]. The blood-retinal barricade, which entails retinal capillary endothelial cells and retinal pigment epithelium (RPE) cells, acts as a protective barrier that confines the admittance of drugs to the later ocular region [27]. Situated between the neurosensory retina and the choroid, the retinal pigment epithelium (RPE) executes a fundamental character in the sustenance of the visual system. It facilitates crucial functions within the visual pathway by preferentially transporting molecules between the photoreceptors and the choriocapillaris. The intercellular permeation is limited by the skintight junctions in the RPE that restrict the passage of molecules between adjacent cells [27]. Although drugs can enter the choroid quickly through oral or intravenous dosing, the superficial blood-retinal barricade confines further admittance into the retina. Overcoming the blood-retinal barricade has been explored through nanotechnology, with studies showing the successful passage of inorganic or metal oxide-based nanoparticles and gene delivery systems [28].

Functionalized nanoparticles, when delivered intravenously, have been shown to target choroidal neovascularization lesions specifically. While specific drugs have demonstrated distribution in ocular tissues following intravenous administration, the utilization of systemic administration for treating ocular disorders is restricted due to concerns related to toxicity and delivery. Researchers have explored oral administration as a non-disruptive and patient-preferred method for administering drugs to the eye [29,30]. This method can be used alone or concurrently with topical delivery methods. Although topical delivery alone may not attain therapeutic concentrations in the retinal region, oral administration presents potential benefits for handling enduring retinal ailments when equated to parenteral routes [31]. The operation of oral sending for ocular drug administration encounters challenges due to restricted access to targeted ocular tissues. Generally, the higher dosages are habitually obligatory to achieve the desired beneficial worth, potentially consequential in non-localized side effects [28]. Safety and toxicity considerations are paramount when seeking a response to therapy in the eye through oral administration [28]. Some drugs, such as oral carbonic anhydrase enzyme blockers used in glaucoma therapy, have been discontinued due to their circulatory venomousness [32]. The use of the insolence pathway for delivering medicines to the eyes is not widely practiced, and the research on drugs for this route is limited [33].

**Fig. 2:**
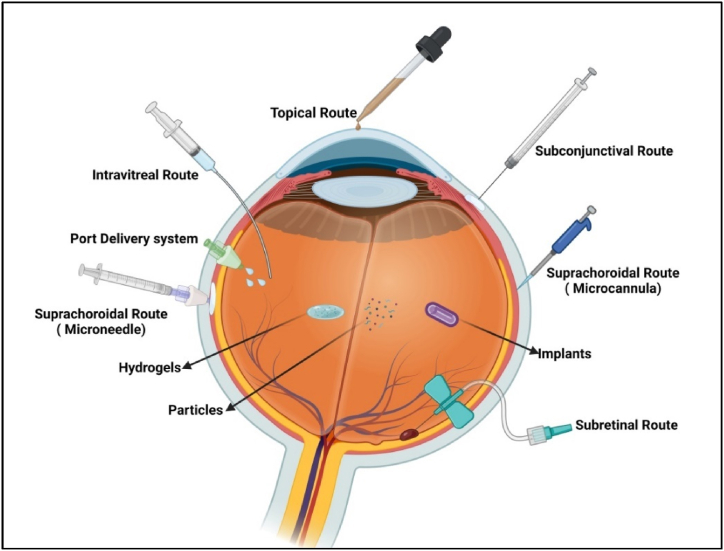
Summarized various advanced routes for ocular drug administrations (Created with BioRender.com).

The periocular and intravitreal administrations are alternative routes to overcome the limitations of mucosal dosing and systemic dosing methods to achieve optimal drug concentrations in the retinal region. These routes offer targeted delivery options for effectively treating ocular conditions. These routes are particularly beneficial in avoiding systemic side effects and catering to geriatric patients [34]. The periocular route comprises techniques such as Sub-Tenon injections, Retrobulbar space injections, and Periocular injections, which are relatively less intrusive than intravitreal injections [34]. Subconjunctival injections bypass the rate-limiting conjunctival epithelial barrier and the cornea-conjunctiva barrier, allowing drug permeation over the sclera and the choroid to spread the neural retina and photoreceptor cells [16]. Intravitreal shots provide the lead of direct drug conveyance into the vitreous humor. Drug distribution within the vitreous can be uneven, with smaller molecules diffusing more rapidly than larger ones. The vitreous humor obliges as a barricade for the conveyance of retinal inheritable influence remedy, as it can impede drug mobility [1,35]. In general, the interactions between negatively charged glycosaminoglycans and cationic complexes can further hinder the movement of drugs. In addition to the vitreous, the inner restrictive crust performs as a barricade that confines the conveyance of medications from the vitreous to the retina. The movement of therapeutics from the vitreous to the distal retinal layers and choroid is a multifaceted process, mainly allied with the RPE [36]. Fig. 2 further summarizes the various routes of the ocular drug delivery system.

It is crucial to determine the therapeutic effectiveness of a drug by understanding its elimination half-life in the vitreous. The elimination process can take different routes, including diffusion into the aqueous humor and passage through the blood-retinal barrier. The half-life of compounds in vitreous humor is extended through their hydrophilicity and higher molecular weight [37]. Various transporters play a critical role in facilitating the transport of nutrients across biological membranes, and they can be used to improve the delivery of ocular medications. Various efflux transporters, i.e., P-gp, MRP, and BCRP lower drug bioavailability by expelling drugs from the cell membrane. Influx transporters are essential in promoting foreign substances and vital nutrients entry into the ocular tissues. The Solute Carrier Family 1 (SLC1), Solute Carrier Family 6 (SLC6), and Solute Carrier Family 7 (SLC7) gene families' unique amino acid and peptide transporters have been identified in ocular tissues and potential targets for drug delivery strategies [38]. Table 2 further summarizes the existence of additional trailers used in transporting ocular drugs, i.e., organic cation/anion, monocarboxylate, and nucleoside trailers. These teasers depict pivotal roles in the absorption and subsequent transport of particular compounds across ocular tissues, which support the comprehensive method of ocular medicine delivery. Prodrugs developed specifically targeting these trailers can increase drug allure, enhance physical and chemical properties, get around efflux pumps, augment drug fascination, improve physicochemical properties, and bypass efflux pumps [38].

**Table 2:** Summarization of various ocular transporters, drugs/prodrugs targeted, and their outcomes for effective drug delivery.

Trailer Arrangement-Targeted	Tissue/Cells	Drug/Prodrugs targeted	Characteristics/Outcomes	Reference
Sodium-reliant neutral amino acid transporter B(0)+	Cornea	l-aspartate ACV	• l-aspartate ACV exhibited a quadruplex advanced Transcorneal perviousness equated to ACV.	[39]
Organic anion transporting polypeptide (OATP) arrangement on the cornea	Cornea	l-valine ACV	• l-valine ACV exhibited a treble advanced trans corneal perviousness equated to ACV.	[40]
		GCV conjugated with Gly-Val GCV conjugated with Val-Val GCV conjugated with Tyr-Val	• The prodrug formulations exhibited notable transcellular passive diffusion and were documented by the trailer, resulting in augmented facts of AUC (area under the curve) and then Cmax (peak concentration).	[1,41]
Organic anion transporting polypeptide (OATP) arrangement on retinal pigment epithelial cells (rPCEC) and the cornea.	Retinal pigment epithelial cells (rPCEC)cells then Cornea	Valine-quinidine Valine-valine-quinidine	• The prodrug formulations were observed to have no recognition by the P-gp efflux pump, indicating that they are not substrates for this transporter. These prodrugs were identified as substrates of peptide transporters, highlighting their interaction with this specific class of transporters.	[42]
OPT system on the retina	Retina	Glycyl-valyl-ganciclovir Valyl-valyl-ganciclovir Tyrosyl-valyl-ganciclovir	• The prodrugs, Glycyl-valyl-ganciclovir, • Valyl-valyl-ganciclovir and Tyrosyl-valyl-ganciclovir, exhibited Double the amount of RCS tissue permeability compared to GCV, which was attributed to their increased lipophilicity and OPT-mediated translocation across retinal pigment epithelium (RPE),	[41]
Sodium-Reliant Multivitamin Transporter (SMVT) on the retina	Retina	Biotin-GCV	• Biotin-GCV demonstrated higher perviousness into the retina-choroid than sluggish purging from the vitreous.	[43]
GLUT1 (Glucose Transporter 1) on the HRPE cells	Human Retinal Pigment Epithelial cells (HRPE) cells	glutamate (Glu) and dopamine	• The prodrug, Glu-dopamine, was recognized by the transporter, indicating its suitability for targeting.	[44]
MRP2 on the cornea	Cornea	Erythromycin	• MRP2 (Multidrug Resistance-Associated Protein 2) demonstrates functional activity alongside P-gp (P-glycoprotein) in actively transporting drug molecules out of the corneal epithelium.	[45]
PEPT1 on the cornea	Cornea	l-valine, l-valine-valine esters of quinidine, val-quinidine (VQ), then val-val-quinidine (VVQ)	• The increased permeabilities observed in the prodrugs suggest that modifying drugs through derivatization can be an effective approach to overcome the efflux mediated by P-gp.	[46]
GLUT1 on the cornea	Cornea	Timolol	• Enhanced corneal penetration, and high enzymatic stability.	[47]

MRP2: Multidrug Resistance-Associated Protein 2, ACV: Acyclovir, RCS: Retinal Pigment Epithelium-Choroid-Sclera, AUC: Area Under the Curve, GCV: Ganciclovir, rPCEC: Rat Primary Corneal Epithelial Cells, SMVT: Sodium-Dependent Multivitamin Transporter, PEPT1: Proton-Coupled Oligopeptide Transporter 1, VQ: Val-quinidine, VVQ: Val-Val-quinidine, GLUT1: Glucose Transporter 1.

Melanin, found in ophthalmic tissues like the RPE and uvea, can interact with medications and change how they are metabolized. Reduced pharmacological efficacy could arise from a considerable reduction in the optimum medication at the effective site due to melanin binding brought about by electrostatic and dispersion forces from London. Preparations that are simple and lipotropic are more inclined to mix with melanin [38]. The binding process is crucial in administering ophthalmic drugs because it could alter therapeutic dosages in the anterior ocular fleshes and affect the projected therapeutic efficacy. Following peri scleral drug administration, choroidal melanin, and RPE impact how therapeutics enter the retina and vitreous. Contrary to the melanin-free sclera, the presence of melanin can lead to a prolonged permeation lag time for lipophilic molecules and reduced solute permeability across the choroid-Bruch's membrane [38].

## 3 Advancement in drug delivery strategies to the anterior segment of the eyes

**Fig. 3:**
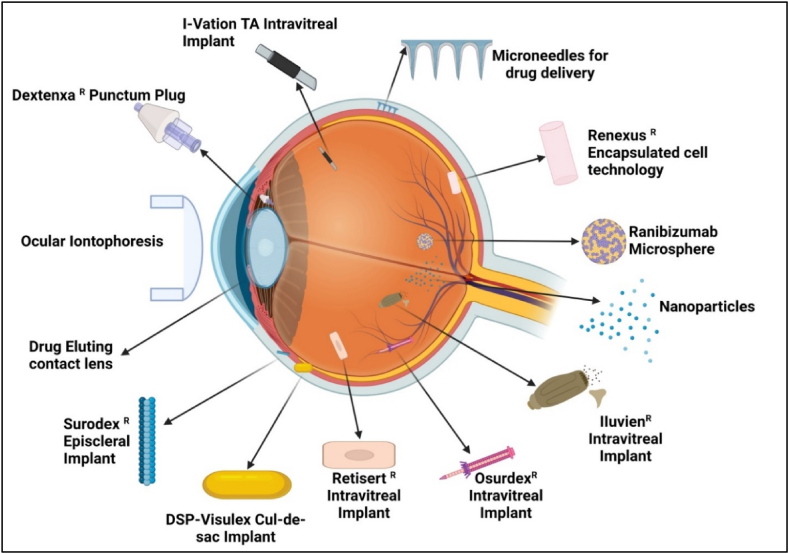
Illustrated the recent advancement in ocular drug delivery approaches (Created with BioRender.com).

Ophthalmic drops are the preferred treatment option for ocular diseases due to their ease of use, accessibility, and non-invasiveness [48]. Various techniques have been employed to enhance the effectiveness of such eye drops. The use of cyclodextrins to improve the aqueous solubility of hydrophobic therapeutics/drugs illustrates advancements in ophthalmic therapy [8,48]. This strategy has resulted in better medication penetration into the eyes. To extend the time that eye drops stay on the ocular surface, viscosity additives, i.e., polyvinyl alcohol (PVA), and cellulose derivatives are generally utilized. These substances improve the efficiency of drug administration without seriously harming ocular cells by lowering drainage and encouraging attraction across the cornea [[48], [49], [50]]. Fig. 3 further describes the various advanced approaches for ocular drug delivery systems in recent years.

### 3.1 Liposomes

Liposomes are specialized colloidal carrier systems that can be utilized for the fore slice of the eye. These microvesicles entail an aquatic core encased in by a hydrophobic lipid layer, allowing for the ease of administration through eye drops. They also offer the advantage of sustained and controlled release mechanisms, reducing the frequency of drug application. When designing liposomes, several factors are taken into consideration, i.e., the negative charge of the corneal mucosa, the functional pH of the eye, drug solubility, molecular mass, lipid bilayer mobility, vesicle integrity, and drug loading efficiency [51]. In a recent year, Dong et al., examined the morphology of liposomes with silk fibroin coating (SLs) for ophthalmic therapy to assess their physical characteristics. The drug encapsulation efficiency of SLs was investigated to determine their ability to contain ibuprofen effectively. The release profile of ibuprofen from solid lipid nanoparticles (SLNs) was investigated through release kinetics analysis in assessment to a drug solution rather than conventional liposome. The learning also appraised the corneal penetration studies of SLs to assess their ability to penetrate the cornea [52]. In addition, Hosny et al., investigated optimizing the incorporation of gatifloxacin in a liposomal aqua gel matrix. Through their investigation, the researchers identified that a specific fraction of phosphatidylcholine, cholesterol, and stearyl amine resulted in the highest permeability. The inclusion of stearyl amine, a positive electrostatic modifier, improved the loading efficiency. Therefore, incorporating carbopol 940 as a hydrogel facilitated sustained and prolonged drug release, enhancing gatifloxacin transcorneal transport and increasing efficiency [53]. In another study, Li et al. investigated the utilization of anionic liposomes coated with modified low molecular weight chitosan (LCH)-loaded diclofenac sodium for corneal delivery. The application of low molecular weight chitosan (LCH)-coated liposomes demonstrated improved average residence time, corneal penetration, and concentration levels compared to uncoated liposomes or DS dissolved in water. The inclusion LCH in the formulation potentially enhanced permeability by interacting with the Cornea having a negative surface charge and potentially tight junction permeabilization. Furthermore, the LCH improved the stability of the liposomes by reducing hydrolysis and oxidation [54].

### 3.2 Microemulsion

Microemulsions are stable mixtures of oil and water that use non-ionic surfactants and co-surfactants. Microemulsions offer several advantages, including transparency, thermodynamic stability, and controlled drug release. Various hydrophobic drugs can be loaded more effectively by formulating microemulsions in different ways, and their residence time in the eye can be extended [55,56]. A study on mucoadhesive chitosan-coated cationic dexamethasone microemulsion system demonstrated that CH-MEs (mucoadhesive chitosan-based microemulsions) give favorable physicochemical characteristics, mucoadhesive solid properties, and excellent stability over three months. The sustained drug-release properties of mucoadhesive chitosan microemulsions (CH-MEs) were also observed. Several *In vivo* experiments on rabbit eyes, utilizing a uveitis-tempted rabbit eye archetypal, displayed a notable upsurge in the anti-provocative worth of the eyes entertained with mucoadhesive CH-MEs Relative to suspension formulation currently in the market [57]. Microemulsion formulations have been found to increase the bioavailability of not just hydrophobic drugs but also non-hydrophobic drugs. For instance, in the case of drugs like pilocarpine, microemulsion formulations have been developed to reduce dosing frequency, resulting in improved controlled release and extended drug retention in the eye [58].

### 3.3 Nanosuspensions

Ocular drug delivery systems utilize colloidal particles falling within the 10–1000 nm size threshold. These particles serve as carriers for entrapping, adsorbing, or encapsulating the drug along with a bio-adhesive polymer for the occurrence of polyacrylic acid (PAA) or its unoriginal [59,60]. Upon application, these nanoparticles agglomerate on the cornea, resulting in an extended retention period on the corneal superficial. This agglomeration augments the diffusion of the non-ionized drug into the cornea, improving drug delivery efficiency. When compared to a viscosity agent like polyvinyl alcohol (PVA), the use of PAA in topical delivery systems increases sustained drug absorption observed at later time points following administration. Sub-micron particles have demonstrated greater absorption compared to larger microparticles [20,21,61]. In research by Adibkia et al., the practice of piroxicam: Endragit® RS100 nanosuspension formulation in reducing inflammation was investigated in rabbits with endotoxin-tempted uveitis (ETU). The nanosuspension construction exhibited superior anti-provocative effects to the microemulsion construction and the unprocessed control set. The nanosuspension's effects lasted up to a 12-h interval, with the maximum therapeutic impact during a 24-h duration. The cationic copolymer, Endragit® RS100, potentially enhanced the residence period in ophthalmic tissues by interacting with negatively charged cells through ionic interactions. This drug-sending scheme has the skill to curtail the required dosage and dosing interval to once daily, which could enhance patient obedience [62].

In recent years, Kassem et al. conducted a study comparing the worth of frequently castoff glucocorticoid therapeutics in nanosuspensions plus micro suspensions in the eyes of rabbits. The nanosuspensions exhibited the highest drug absorption and maximum intraocular pressure (IOP) reduction, indicating superior efficacy. This research suggests that the claim of nanoparticle suspensions in innovative ocular drug-sending structures could enhance patient adherence by offering once-daily dosing options [63].

### 3.4 Ocular inserts and minitablets

The specialized hard or semi-hard wafers or tablets created specifically for ophthalmic uses are called ocular inserts plus minitablets. The front of the eye segment and cornea can get continuous drug delivery thanks to the placement of these inserts in a cul-de-sac [64]. They provide numerous benefits, such as changed therapeutics pharmacokinetic profiles and clinical outcomes related to extended exchange length and ongoing drug announcements. They can lessen systemic toxicity and reduce the frequency of instructions, improving patient adherence. Ophthalmic inserts made of soluble hydroxypropyl cellulose have been tested and proven to reduce the symptoms of dry eyes significantly. However, these inserts can occasionally cause extraneous-build consciousness, discomfort, and blurred vision, possibly leading to the end of therapy [64].

To improve the ocular administration of medications, ocular bio-erodible minitablets, which are similar to ocular inserts, were developed and used. The insertion of these minitablets into the fornix of the eye has been shown to increase gentamicin delivery for treating infectious keratitis [65]. Ciprofloxacin minitablets without preservatives are considered bioequivalent to Ciloxan® eye drops used on a 30-min basis because they have been demonstrated to maintain bactericidal levels in the tear film for up to 8 h [66]. Another novel delivery method is Ophthacoil, which uses a metallic wire wound around an aqua gel coating containing a medication. When the hydrogel comes into touch with tears, it expands, enabling gradually controlled medication release. Ophthacoil displayed persistent pradofloxacin problems over the minimal inhibitory concentration (MIC) for 16 h in a trial on dogs [67].

### 3.5 Ocular implants for the anterior chamber

Surodex™ is a bioerodible matrix implant developed by Allergan Inc., specifically designed to release 60 μg of dexamethasone over 7 days. The implant entails a rod-fashioned polymer matrix finished with PLGA (poly (lactic-*co*-glycolic acid)), HPMC (hydroxypropyl methylcellulose), and dexamethasone [68,69]. It is placed within the front eye chamber and serves as an anti-provocative negotiator for the affected role undergoing cataract surgery. Surodex™ is currently undergoing late-stage scientific hearings and has demonstrated good patient tolerability. However, its effectiveness appears comparable to topical steroids, as distinguished in the learning [70]. Punctal plugs, also denoted as punctum plugs or lacrimal plugs, also occluders, are small biocompatible inserts used for sustained ophthalmic therapy [71]. These plugs are typically the size of a rice grain and can be inserted into the lacrimal duct. They come in various designs and shapes, often made of silicone, collagen, hydrophobic acrylic polymer, aqua gel, and polydioxanone. Once inserted, intracanalicular plugs may adopt a semi-hard state or expand to block the cavity. An advantage of this ocular device is its ease of insertion in elderly patients due to age-related enlargement of the puncta [72].

## 4 Advancement in drug delivery strategies to the posterior segment of the eyes

The treatment of ocular disorders that mainly affect the back part of the eye, like diabetic macular edema, senile macular degeneration, and posterior uveitis, poses significant difficulties when relying on traditional methods of topical or systemic drug delivery. The issue is mainly caused by ocular vascular barriers that prevent drug absorption into the eye tissue [1]. The aquatic humor fence and the retinal vascular barricade protect the subsequent slice in addition to the corneal barrier already stated. The blood and intraocular fluid, flanked by the anterior eye barrier and created by the ciliary body, respectively, play a vital part in maintaining homeostasis. Similarly, the neurovascular retinal barrier controls the vitreous humor chemicals' removal while allowing access to a crucial metabolic substrate. Because of these obstacles, researchers have been forced to consider and create other methods of treating diseases that affect the latter part of the eye [1,19,73,74].

### 4.1 Sustained release ocular drug implants

Various sustained-release ocular drug implants have proven effective in treating chronic ocular ailments that distress the later eye sector, such as uveitis, cytomegalovirus (CMV) retinitis, and neovascular age-linked macular deterioration. These implants, whether non-biodegradable or biodegradable, offer significant advantages by bypassing the blood-ocular barrier, providing extended drug delivery, and minimizing toxicity [75,76]. Non-biodegradable implants, including Retisert®, Vitrasert®, and Iluvien®, have been developed explicitly for specific indications. Retisert® is designed for CMV retinitis, Vitrasert® for idiopathic uveitis, and Iluvien® for diabetic macular edema (DME) [77,78]. This surgical implantation into the later eye sector distributes the treatment over an extended period. However, they can also possible adverse reactions and complications like retinal detachment and endophthalmitis. Iluvien® has demonstrated positive results in clinical trials for DME, with significant visual improvements lasting over 30 months [79].

Nevertheless, specific safety issues highlighted by the FDA have led to the request for additional clinical trials [56]. Biodegradable ocular implants utilize a matrix composed of biocompatible polymers, for illustration, polylactic acid (PLA), polyglycolic acid (PGA), and otherwise polylactic co-glycolic acid (PLGA), along with the drug. These implants are converted into harmless metabolites within the body and offer various advantages. They do not entail deletion once the drug is depleted from the implant. Ozurdex® is an illustration of a green scion operating a dexamethasone intravitreal PLGA scion to handle macular edema and then non-infectious uveitis [57]. It employs the NOVADUR™ platform to sustain drug release, initially showing a burst followed by slower diffusion and polymer degradation. Innovations in ocular drug delivery include non-polymer-based systems like Verisome™ developed by Icon Bioscience Inc. The system is injected into the target site using a needle and degrades simultaneously with drug release without causing toxicity [76].

### 4.2 Gene therapy

Gene therapy refers to the inherited quantifiable conveyance, for illustration, DNA or RNA, into target cells to manipulate their inheritable influence expression. Initially, viral vectors delivered gene-modifying nucleic acids, allowing for stable expression within target cells [80]. However, viral vectors have limitations, including immunogenicity and the need for invasive administration methods. Different growth factors have been targeted in ocular gene delivery, and two FDA-approved drugs, Intraocular implants® and Macugen®, have been developed. Vitravene® is used to treat human cytomegalovirus (HCMV) in AIDS patients, while Macugen® is used for wet age-interrelated macular deterioration. These drugs bind to specific targets, inhibiting viral replication or suppressing ocular neovascularization [80]. For minimally invasive alternatives, RXi Pharmaceuticals has partnered with EyeGate Pharma to explore using electro-assisted, non-invasive drug transport technology for administering sd-rxRNA™ (self-delivering rxRNA) [81]. Spark Therapeutics is in early-phase clinical trials and Phase III trials for choroideremia and Leber congenital amaurosis, respectively [82]. Avalanche Biotechnologies is in Segment II hearings for neovascular age-interrelated macular deterioration [83]. Oxford BioMedica, in collaboration with Sanofi, is conducting Phase I trials for Stargardt ailment and Segment I/II hearings for Usher syndrome [84]. These advancements in gene therapy hold promise for addressing these debilitating ocular conditions.

### 4.3 Drug-eluting punctal plugs

Typically, most of the Food and Drug Administration (FDA) approved drug-eluting punctal plugs block tear drainage when inserted into eyelid lacrimal puncta. These plugs, typically made from polymers, have traditionally been used to treat dry eye disease. Companies such as Mati Therapeutics and Ocular Therapeutic are developing them as sustained drug delivery systems for various ocular conditions. Mati Therapeutics is currently engaged in the clinical development of a punctal plug called Evolute latanoprost. This plug releases latanoprost over three months to treat wide-angle glaucoma and elevated intraocular pressure (IOP) [85,86].

On the other hand, Ocular Therapeutic is evaluating their swellable, degradable hydrogel plugs that are impregnated with drugs like dexamethasone for post-operative inflammation and pain, as well as timolol for wide-angle glaucoma or elevated intraocular pressure (IOP) [87]. These drug-eluting punctal plugs address the challenge of patient compliance by providing sustained drug release. They can be customized to enhance therapeutic effectiveness while reducing side effects. The insertion of these plugs is a simple office procedure performed by eye care professionals. This approach can optimize drug delivery, resulting in improved treatment outcomes for patients with various ocular conditions [86].

### 4.4 Vascular targeted photodynamic therapy (PDT)

Vascular Targeted Photodynamic Therapy (PDT) is a handling repetition operated in ocular drug therapy, with Visudyne® being the sole FDA-authorized ocular drug incorporating PDT. Visudyne® consists of the photosensitizer compound verteporfin formulated in liposomes. The treatment involves IV administration of the photosensitizer, followed by non-thermal laser irradiation of the retina [88]. Upon laser activation, the photosensitizer generates cytotoxic free radicals that selectively target neovascularization, causing the blockage of the targeted blood vessels. However, Visudyne® has been found to originate hurt to retinal pigment epithelium (RPE) cells and then photoreceptor lesions [88,89]. Steba Biotech S.A. is conducting Segment II medical hearings to investigate the well-being and treatment outcomes of Stakel®-based vascular-directed photodynamic therapy for the affected role with choroidal neovascularization allied with age-linked macular degeneration (AMD) [90]. This ongoing research aims to evaluate the latent of Stakel® to illustrate an alternative treatment option for this specific ocular condition and assess its effectiveness and safety compared to existing therapies [90].

### 4.5 Intraocular implants

Commercialized ocular implants made with modified polymers and a coating on intraocular lenses (IOLs) can help overcome complications after cataract surgery, including posterior capsule opacification [91].

#### 4.5.1 Blue light-blocking lens

According to a patent from 2015, a combination of a monomer used in the production of plastic lenses and a compound consisting of thiophene and benzene can be advantageous for eye protection by dispersing blue light. In addition to the material dispersing blue light, the mixture may include an ultraviolet light-blocking substance, a pigment, and a polymerization initiator, which are further mixed into the monomer. This formulation aims to provide enhanced protection against harmful blue light and ultraviolet radiation while incorporating other beneficial properties for eye health [92].

#### 4.5.2 MicroShunt minimally invasive glaucoma surgery

The RESERFLO®/MicroShunt is a glaucoma treatment device with a polymer dubbed poly(styrene-*block*-isobutylene-*block*-styrene) (SIBS). This stratagem is designed for minimally invasive glaucoma surgery (MIGS) and offers some reduction in intraocular pressure (IOP) and relief from glaucoma medications [92]. However, one limitation of the MicroShunt is its relatively large plate size. To overcome this challenge, there is a requirement for a compact glaucoma drainage device that does not rely on plates [92]. The device should be constructed from a polymer material that maintains its integrity over time and does not contribute to the development of substantial scar tissue or encapsulation. Researchers are exploring alternative materials and designs to develop more compact and efficient glaucoma drainage devices with improved long-term outcomes [92].

#### 4.5.3 INVITE C in diabetic retinopathy

The curcumin is loaded in polymer inulin-d-α-tocopherol succinate bio couples (INVITE), so the combination is called INVITE C. INVITE C nano micelles have shown promise in intraocular therapy for diabetic eye diseases, including diabetic retinopathy [93]. These nano micelles possess antioxidant activity that aids in protecting human retinal pigment epithelium cells, particularly under high glucose conditions. This characteristic enhances their potential as a therapeutic option for diabetic eye diseases [93]. There is still a constant search for polymers that are biocompatible and biodegradable and have better results and preclinical and clinical trials. The usage of modified polymers assured more residency time for eye drop formulation. Despite the ongoing challenges in corneal penetration, there is still significant potential for further exploration in the field of ophthalmic therapies using polymers. Combining polymer science with interventional studies in preventive and therapeutic approaches in ophthalmology has opened up new possibilities for the future of this field [23].

Table 3 highlights the need for alternative patient-compliant delivery systems and optimizing the potency of drug therapy for ophthalmic medications before administration.

**Table 3:** Technological advances in Ocular drug delivery.

Device	Application	Manufacturer/Year of Launching or Approval	Advantages	Limitations	References
VersiDoser™ Ophthalmic Delivery System	Ocular drug delivery	Mystic Pharmaceuticals®/2006	Precise dosing, aseptic, preservative-free	Multiple daily administrations required	[22]
TrueTear® Tear Neurostimulator	Dry eye treatment	Allergan/2018	Non-invasive stimulates tear production	Limited to dry eye treatment	[94]
Eyenovia Optejet™ Dispenser	Ocular drug delivery	Eyenovia, Inc/2023	Micro-dosing technology, targeted delivery	Self-administration may have a learning curve	[95]
iDose TR Controlled-Release Implant	Sustained drug delivery	Glaukos Corp/2023	Extended drug release, improved compliance	Requires implantation procedure	[93]
Nano-wafer Technology	Ocular drug delivery	–	Convenient, controlled release	Limited to certain types of medications	[96]

## 5 Excipients castoff in ocular inventions for modified drug conveyance

### 5.1 Viscosity increasing polymers

Enhancing the viscosity of the vehicle is a technique used to extend the residence time of ophthalmic drugs [97]. Synthetic polymers such as polyethylene oxides, polyacrylates and polyvinyl alcohols, polyesters, polyolefins, collagen, gelatin, and dextran are used to accomplish this increase in viscosity. Viscoelastic eye drops, on the other hand, may promote reflex tearing, resulting in faster medication clearance. More significant viscosity eye drops irritate patients, sometimes do not allow for repeatable drug doses, and produce blurred vision after delivery. An ideal viscosity range of 15–55 P (P) is thus advised, allowing for a longer residence time and avoiding adverse effects, as previously stated [98].

#### 5.1.1 Polyethylene oxides

Polyethylene glycol (PEG) is a colorless, transparent hydrophilic polymer derived from ethylene oxide monomers, which can improve the solvability, biological compatibility, and efficacy of the therapeutic moiety. PEG has various configurations (e.g., multi-armed linear) and molecular weights. The FDA considers it to be generally safe (GRAS) and has approved it for various purposes, including ophthalmic usage [99]. Short-acting implants, like Dextenza®, a PEG-based tubular implant, make use of PEG's controlled release features by preventing erosion by hydrolysis to give 1 month extended release of dexamethasone for both pain and inflammation control following surgery [100]. PEG is also used in other Ocular Therapeutix medications, including intracanalicular ocular insert of dexamethasone (OTX-DED) and tyrosine kinase inhibitor. OTX-DED is a low-dose, short-stretch therapy that uses the identical PEG technologies as Dextenza to deliver dexamethasone [101,102].

Thermosensitive polymers have received the most significant attention in terms of administration. To be appropriate, a thermogelling polymer must possess a gel formation temperature in an array of 32–36 °C so that it is molten at room temp but performs an immediate conversion to gel over the ocular shallow [101]. These polymers can be natural and synthetic. Poloxamer 407 is the most often used and readily available synthetic polymer. The general formula is PEOx- PPOy-PEOx, and it is made up of polypropylene oxide (PPO) and polyethylene oxide (PEO) units [103]. To enhance the drug tenure of dwelling in the precorneal region, a blend of mucoadhesive polymers and situ gelling polymers with high cohesive and mucoadhesive qualities looks favorable [104] like chitosan/poloxamer [105], polycarbophil/poloxamer [106], carboxymethyl cellulose/poloxamer [107], polyacrylate/poloxamer [108], gellan gum/poloxamer/polyacrylate [108] or hydroxypropylmethycellulose/poloxamer [109]. The DuraSite® system appears acceptable as a specimen of commercially accessible eye drops incorporating this combination [110]. It shows a temp-sensitive sol-gel conversion due to poloxamers and excellent much-gummy features due to a polyacrylate. A second variant of this arrangement (DuraSite 2®) was created in another study. Adding a positive-charged polymer to the current generation of eye drops ensured an extended dwelling period of the therapeutics in the precorneal precinct. These podia were utilized in the production of eye drops of azithromycin (AzaSite®) [111], and moxifloxacin (Besivance®) [112]. In the study, Lakhani et al. demonstrated that incorporating PEG (polyethylene glycol) into nanostructured lipid carriers enabled the anti-mycotic drug amphotericin B solubilization. This formulation showed potential for ocular antifungal topical treatment. PEG is currently being investigated in clinical trials for its therapeutic PEGylation properties [113].

#### 5.1.2 Polyvinyl alcohols (PVA)

Polyvinyl alcohol (PVA) is a synthetic polymer that is biodegradable and water-soluble. It has various applications in polymer manufacturing, medicine, and nutrition industries. PVA (polyvinyl alcohol) is frequently employed to enhance the drug's solubilization in aqueous media because of its resistance and processability. It has a low acute oral toxicity, with an LD50 (median lethal dose) ranging from 15 to 20 g/kg [114]. Furthermore, PVA is poorly absorbed through the digestive tract. The unique structure of PVA allows for variable permeability, making it suitable for sustained formulations. The recent approval of Yutiq®, an implant composed of polyimide/PVA, further expands the possibilities for controlled drug sending, mainly for handling uveitis. PVA is also painstaking for superficial ocular medication healing using wafer formulations. For illustration, a study by Dipak et al., developed PVA nanofibers loaded with gatifloxacin, demonstrating prolonged precorneal contact duration and a two-step announcement rough draft with a rapid preliminary sector tailed by the continual announcement for up to 7 h. This extended-release profile reduces the prerequisite for recurrent direction in treating parched eyes [115]. Similarly, Javid et al., demonstrated that utilizing nanofibers placid of levofloxacin-conjugated chitosan incorporated into PVA showed improved sustained release characteristics. The findings highlight the effectiveness of drug conjugation to the polymer core in reducing burst release and achieving prolonged release of levofloxacin [116].

#### 5.1.3 Polyesters

The artificial polymers PLA (polylactic acid), PGA (polyglycolic acid), and PLGA (poly (lactic-*co*-glycolic acid)) are frequently used in ocular preparation conveyance arrangements. The FDA has given clearance to these hydrophobic polyesters for use in ocular applications. Lactic acid is the source of PLA, whereas glycolic acid is the source of PGA [117,118]. Both are then generated by ring-inaugural polymerization. PGA breaks down more quickly than PLA because it is more hydrolyzable [117]. The phase 2 clinical trials for slow-release brimonidine in treating geographic withering associated with age-linked macular degeneration were conducted on Brimo DDS®, an intravitreal implant comprising PLA [119]. Based on the monomer ratio and end groups, PLGA, a copolymer of PGA and PLA, offers configurable biodegradation properties. It enables better bioavailability, increased biocompatibility, and controlled release of medicines. The adjustment of crystallinity, degradation time, and hydrophobicity is possible by adjusting the PGA/PLA ratios. The FDA-approved intravitreal implant Ozurdex® uses PLGA to release dexamethasone gradually over 4–6 months before entirely disintegrating in the body. The FDA has approved Durysta®, another PLGA-based implant, for IOP-lowering therapies in wide-angle glaucoma patients [120,121]. Researchers have looked into several modifications and polymer combinations in the search for aesthetically pleasing ocular drug conveyance. For treating inflammation, PLGA-containing nanoparticle formulations, in combination with other excipients, demonstrated therapeutic advantages, tolerability, and better corneal penetration. Additionally, PLGA nanocore lipid polymer nanoparticles have been demonstrated to boost corneal penetration, prolong drug release, and enhance therapeutic advantages [121].

#### 5.1.4 Polymethacrylates

Poly (methyl methacrylate) (PMMA) then added methacrylate-grounded polymers are acrylic, green thermoplastics acknowledged for their water transmissibility, mechanical strength, and thermal stability [122]. These polymers have been used in ophthalmic applications since 1936 and have gained FDA approval for intraocular use [77]. Methacrylate-based polymeric biomaterials have been somewhat cast off in ocular healings, plus the development of nanoparticles, micelles, ocular implants, and hydrogels for equally topical conveyance than intravitreal shot. Methacrylate derivatives such as poly(2-(dimethylamino) ethyl methacrylate) (DMAEM) and poly (2-hydroxyethyl methacrylate) (HEMA) have been investigated in various studies. For instance, PMMA (polymethyl methacrylate) has been utilized as a non-degradable substance coating in intravitreal implants like I-vacation, allowing for extended release of triamcinolone acetonide to extravagance diabetic macular edema [123]. HEMA and other methacrylates have been explored to develop atorvastatin-eluting lenses to manage ocular disorders associated with diabetes [124]. Poly(amidoamine) (PAMAM), a dendrimer, has received approval from the FDA for specific applications. However, its inclusion in the Generally Recognized as Safe (GRAS) list is limited due to toxicity concerns. PAMAM has been studied with dexamethasone for extended drug release in the posterior eye for diabetic retinal dysfunction and macular impairment. PAMAM conjugation to fluocinolone acetonide has been investigated for managing retinal vasculitis in conditions like macular impairment and retinal pigmentary degeneration [125]. These studies demonstrated the effective conjugation of therapeutic agents to the dendrimer, which was given to rats via intraocular injection.

#### 5.1.5 Polyolefins

Poly (acrylic acid) (PAA), acknowledged by its commercial name Carbopol®, is a non-natural polymer made up of acrylic acid monomers. It exhibits excellent aquatic miscibility and thickening capabilities [126]. While PAA is biodegradable, it is imperative to note that the metabolites of acrylic acid can potentially induce inflammation. PAA carries a charge in physiological environments, contributing to its favorable mucoadhesive properties. In experimental hydrogel applications for anterior ocular delivery, Poly (acrylic acid) (PAA) has been combined with other polymers, such as PNIPAAm, to create temperature-sensitive in situ hydrogels. These have been employed for sustained release of epinephrine, particularly in treating glaucoma [127]. PAA has received FDA approval for various epidermal applications, including ophthalmic delivery. It is marketed in different eye drop formulations. One is Restasis®, an ocular emulsion that utilizes a carbomer copolymer sort A-grounded arrangement to convey cyclosporine in managing parched eye settings [128]. In research by Moustafa et al., fluconazole liposomal hydrogels were developed and synthesized as innovative arrangements to enhance ocular remedy conveyance and prolong the dwelling time. The results demonstrated that these liposomes exhibited an extended dwelling stretch in the eye, lasting more than 1080 min [129].

#### 5.1.6 Collagen

A fibrous protein called collagen is found naturally in many connective tissues, such as the sclera, cornea, lens capsule, and vitreous humor. Collagen is readily available from mainly bovine and porcine origins as it occurs naturally, is enzymatically degradable, biocompatible, and relatively simple to make. Recombinant collagen is now widely available, which has decreased reliance on animal sources for collagen manufacturing. Plant and yeast cells can produce recombinant collagen, which has benefits such as increased uniformity and safety throughout production [130]. Following ocular injury or cataract surgery, collagen shields have been used to protect the eyes for a long time. In various methods, it has continued to be used as a drug delivery system in ocular research. It has been utilized for encapsulated cell treatment, in which collagen encases cells and delivers them intravitreally. Collagen-based gels have been used to achieve extended drug release.

Similarly, collagen has been utilized to achieve the same effect. Recent research has implemented it as a platform for regenerating retinal tissue, demonstrating its promise for tissue engineering uses in the retina [126]. Photrexa® is a contemporary ocular medicine delivery system that uses collagen [131]. A riboflavin eye solution filled with collagen crosslinks the biopolymer when subjected to ultraviolet A light.

#### 5.1.7 Gelatin

Gelatin is a proteic polymer fashioned by the irreversible process of collagen breakdown. It is biodegradable, non-toxic, viscosity boosting, gel-forming, readily available, and low in cost, yet it has the advantage of a lower gel formation temperature and better aqueous solubility [102,132]. It is GRAS-certified and produced from avian, mammalian, and ichthyoid collagen I sources, enabling a wide variety of accessible molecular weights. One can use recombinant gelatin with specific molecular masses and isoelectric points to minimize immunogenicity. Gelatin has been used in ocular drug administration in eye drops to act as demulcent, posteriorly and anteriorly administered hydrogels, nano subdivisions for continual announcement, ophthalmic flesh manipulation, and then siRNA carter for inherited healing [23].

#### 5.1.8 Dextran

Dextran is a polysaccharide biopolymer placid of d-glucose slices and is lactic acid bacteria-produced dextran. Dextran can create hydrogels and serve as a carrier for siRNA in inheritable influence healing claims [133]. Dextran is a biopolymer authorized by the FDA for use in ocular eye drops like Tears Natural II® and Tears Natural Forte® as treatment options for dry eye [134]. It is conveniently chemically crosslinked and is being tested for intravitreal and topical administration of ocular therapies with chitosan, PLGA/PLA, then PEG. An experimental study demonstrated the effective administration of lutein, an antioxidant, using crosslinked dextran-chitosan nano subdivisions for topical claim [135]. Dextran can also be used to conjugate drugs for ocular administration. With a low molecular weight, Dextran has been tested as a carrier of cationic DNA, enabling targeted gene therapies for managing X-linked juvenile retinoschisis. Following intravitreal injection of rats, researchers successfully transfected and expressed a complex of dextran-protamine-DNA deposited over the surface of solid-lipid nanoparticles [23].

### 5.2 Mucoadhesive polymers

Mucins comprise no fewer than 20 anionic O-glycosyl proteins, making up the membrane gel layer that protects the eye surface. Mucins create a glycocalyx layer on the conjunctival and ocular surfaces [136]. Excipients that provide adherence to this mucous membrane have a long ocular residence duration [137]. To enhance mucoadhesive properties, polymers can possess specific structural elements. These include: (i) Strong Charges: Polymers with robust charges promote ionic interactions, contributing to mucoadhesion. (ii) Hydrogen Bonding Groups: Functional groups like carboxyls, hydroxyls, amino, and sulfate groups have the skill to fashion robust hydrogen ties. These assemblies facilitate mucoadhesion. (iii) Molecular Weight and Chain Flexibility: Polymers with high molecular weight and chain flexibility facilitate crosslinking with the mucus membrane. This enables the formation of chain entanglements, enhancing mucoadhesion. (iv) Favorable Surface Energy: Polymers with surface energy that supports the spreading of mucus enhance their contact with the mucosal surface, improving mucoadhesion. These structural elements collectively contribute to the mucoadhesive properties of polymers, enabling effective interaction with mucus membranes. This interaction is crucial for applications such as drug delivery systems [138]. Mucoadhesive polymers must cling to the ocular mucus membrane while simultaneously releasing the medicine coupled to the polymeric chains.

#### 5.2.1 Cellulose derivatives

Cellulose derivatives can be categorized into cellulose ether and cellulose ester. Cellulose ether includes hydroxypropyl methyl cellulose (HPMC), methylcellulose (MC), carboxymethyl cellulose (CMC), ethyl cellulose (EC), hydroxyethyl cellulose (HEC), then hydroxypropyl cellulose (HPC). In contrast, cellulose ester comprises cellulose acetate phthalate (CAP) plus cellulose acetate (CA) [139]. Among these derivatives, carboxymethyl cellulose (CMC) is a commonly used polysaccharide as it exhibits improved water solubility due to the incorporation of carboxy groups into the biopolymer chains. CMC often appears in topically delivered eye solutions such as Optive® or Refresh® in managing dry eye owing to its hydrophilicity and biocompatibility. However, numerous additional brands and formulations are available [96]. In an investigation by Yuan et al., CMC-based Nano wafers were created and characterized for prolonged anterior drug administration axitinib. Compared to typical eye drop delivery, the topically administered transparent Nano wafers feature therapeutic nano reservoirs that provide extended drug release and enhanced bioavailability [140].

The initial cellulose polymer to be developed was methylcellulose more than 60 years ago. Methylcellulose has no taste or odor, is water soluble, and is non-toxic. As a result, cellulose ethers are frequently employed in eye drops as viscosity-increasing agents and for wetting properties, which can shorten connection times due to their crust-establishing properties [141]. Cellulose spin-offs have LCST (lower critical solution temperature) behavior, which is a temperature-dependent sol-gel phase conversion, and the gelation process involves hydrophobic interactions between molecules with methoxy substitution [142]. This phase transition feature provides the ability to apply thermoresponsive ophthalmic therapy. Electrolytes, sugars, surface-energetic agents, and natural gums can all affect gelation by lowering the gelation temperature and reducing polymer hydration. An increase in the concentration of methyl cellulose results in a linear reduction in gelation temperature [139].

Hydroxypropyl methyl cellulose (HPMC), also acknowledged as Hypromellose is a hydrophilic polymer with a white or pale white appearance. It finds various applications as a meticulous announcement mechanism in oral and oro-mucosal drug conveyance. HPMC has the skill to swell and then fashion a gel, and it exhibits stability within the pH range of 3–11. It is resistant to gastric enzymes and is commonly used as an excipient for extended-release formulations and thickening, emulsifying, and stabilizing purposes [143]. HPMC, a cellulose-based semi-synthetic dietary fiber, consists of anhydrous glucose units. When exposed to water or gastrointestinal fluids, it forms a viscous solution. HPMC is utilized to enhance the permeation of hydrophobic therapeutic molecules, thereby improving their bioavailability. Liu et al., conducted a study on the ocular administration of FK506-encumbered nano micelles composed of amino-finished polyethylene glycol block poly (D, l)-lactic acid, then HPMC. This study demonstrated the boosted pharmacokinetics and therapeutic worth of FK506 in treating ocular athwart-allograft rebuff [144]. Nanda et al., inspected the enhancement of corneal permeability for the anti-inflammatory drug amlodipine using HPMC. They examined the upshot of sulphobutyl ether-cyclodextrin on a rabbit archetypal and then found that sulphobutyl ether-cyclodextrin improves the permeability of amlodipine-HPMC film [145].

#### 5.2.2 Hyaluronic acid (HA)

In various tissues, Hyaluronic acid (HA) is a glycosaminoglycan with a dominant character in the extracellular matrix. It possesses the skill to bind aquatic jots over hydrogen connection, which augments the stabilization of the lacrimal film and reduces the blinking reflex. However, HA-based ophthalmic formulations typically have a brief duration of ocular residence despite the influence of molecular weight on mucoadhesiveness. It has been observed that higher molecular weight HA exhibits greater mucoadhesiveness [146]. In a study by Salzillo et al., six commercially available formulations, including Blugel®, Bluyal®, Artelac Splash MDSC®, Hyabak®, Octilia Natural®, and Hyalistil Bio®, were compared to newly formulated HA-based eye drops. Among them, Bluegel® demonstrated enhanced fluid drainage grounded on nix-shear viscosity optimization of 24.2 mPas. This was selected as the bull's eye worth for consequent optimization trials [109]. In addition, Liu et al., also demonstrated the transplantation of retinal precursor cells consuming an HA-grounded hydrogel for accurate placement within the sub-retinal region without impairing their functionality. After complete HA degradation, the cells unveiled mature photoreceptor pointers [147].

Additionally, HA has been operated as a self-regenerating internal needle glaze for intravitreal injections to curtail drug spillage beyond the eye [148]. HA is commonly used as a lubricant in parched eye drops, acting stimulated tear crust in harvests for illustration, Vismed Multi, Optive Fusion, Hyalistil®, DROPSTAR®, then Neop. SolarazeTM gel is another application where HA gel is used to create a diclofenac prolonged-release formulation, providing management of eye inflammation and soreness [23]. Researchers have developed a thermosensitive in-situ hydrogel using HA and poly(N-isopropyl acrylamide). This hydrogel, incorporating ketoconazole as an active ingredient, has demonstrated enhanced therapeutic efficacy while minimizing adverse effects in *In vivo* trials [149].

#### 5.2.3 Chitosan

Chitosan is a green biocompatible polymer obtained by deacetylating chitin. It shows promise as an additive in ophthalmic formulations because its reactive amino clusters can intermingle with the cornea and then conjunctiva. However, unmodified chitosan is insoluble at physiological pH, particularly pH values above 5, which limits its topical application in ocular formulations [4]. To address this challenge, various spinoffs of chitosan have been developed for illustration N-carboxymethyl chitosan, then quaternary ammonium-chitosan like N, O-[N, N-dimethylaminomethyl (diethyldimethylene ammonium)n]methyl chitosan or N-trimethyl chitosan (TMC) [150]. While chitosan has limited FDA clearance, it is not approved for ocular use. Pharmacokinetic studies suggest efficacy [4].

Chitosan liposomes and micelles are skilled in carrying a high preparation payload and providing extended medication announcements, making them suitable for intravitreal injection. Chitosan is extensively exploited as a polymer encapsulation for less biocompatible anionic polymers in the step-by-step fabrication of core-shell biomaterials, for transporting anionic medications, and then inherited things owing to its cationic properties [151]. Recent research has focused on chitosan-based hydrogels to enhance the bioavailability of topically applied antibiotics like levofloxacin. Temperature-sensitive hydrogels containing hexanoyl glycol chitosan demonstrated reduced ocular discomfort and 1.92-fold advanced worth in rabbit aquatic humor equated to conventional antibiotic suspensions [152]. Researchers have developed glucocorticoid-containing solutions, such as an aquatic-unsolvable methyl-cyclodextrin-ammonium chitosan combination, which exhibits mucotackiness and cytocamaraderie [153].

#### 5.2.4 Guar gum

Guar gum is a biopolymer consequential from seeds that consist of rectilinear spine chains of β-d-mannose parts with branching points of α-d-galactose parts. It is a biocompatible, water-soluble polymer with viscosity enhancement, non-ionic nature, mucoadhesion, and hydrolytic degradability [23]. Due to its gelling properties, guar gum is commonly used in moisturizing eye drops and has received FDA approval for ocular repetition. It is sparingly soluble in certain solvents and alcohol-containing formulations, and its stability in solution can be challenging. To address these limitations, derivatives, including hydroxymethyl-guar gum, *o*-carboxymethyl *o*-hydroxypropyl-guar gum, and hydroxypropyl-guar gum, have been fashioned to rally solvability and permanency [154]. Hydroxypropyl-guar gum is often incorporated into lubricating eye drops [155]. In experimental studies, guar gum is being explored to enhance the bioavailability of natamycin, an antifungal agent used to manage ocular fungal contagions. This is achieved by incorporating guar gum into polyethylene glycol (PEG) nano-lipid carriers, which enable controlled release through a gelling system integrated with carboxy vinyl polymer and borate [156]. Guar gum-based micelles composed of poly(ε-caprolactone) (PCL) are being studied for extended release of ofloxacin, an antibiotic. These micelles and biotinylated glutathione, retinol, and cell-selective targeting molecules have shown continual medicine announcements for the tiniest 8 h [157].

#### 5.2.5 Pullulan

Pullulan is a polysaccharide receipted from the yeast Aureobasidium pullulans, entailing maltotriose slices connected by α-1,6 glycosidic bonds. It possesses several beneficial properties, including being non-ionic, biocompatible, stable across a wide range of temperatures then pH assortment, aquatic miscible, non-solvable in most organic diluters, effortlessly manageable, oxygen barricade, viscidness-boosting, then environmentally degradable [158]. It has received Generally Recognized as Safe (GRAS) certification from the FDA and then finds applications in various biopolymer-based systems, including ocular drug delivery. Modification techniques like sulfation or amination are often employed to introduce charges and enhance their reactivity due to their non-ionic nature. Co-polymerization of pullulan with synthetic polymers or other biopolymers has publicized talent in outspreading the bio dilapidation frequency equated to using pullulan alone. For instance, the combination of pullulan and gellan gum has been utilized to create electrospun nanofibers for developing in-situ gels, enhancing the bioavailability of topically administered therapeutics.

The gelation assets of pullulan in aquatic make it apt for applications such as hydrogel inserts and thin films. A study by Pai and Reddy evaluated a 10 % pullulan gel insert designed for conjunctival preparation direction for its *In vitro* characteristics. The insert exhibited a complete breakdown and preparation announcement within 3 h of the claim in the *In vitro* setup [159].

#### 5.2.6 Cyclodextrins

**Fig. 4:**
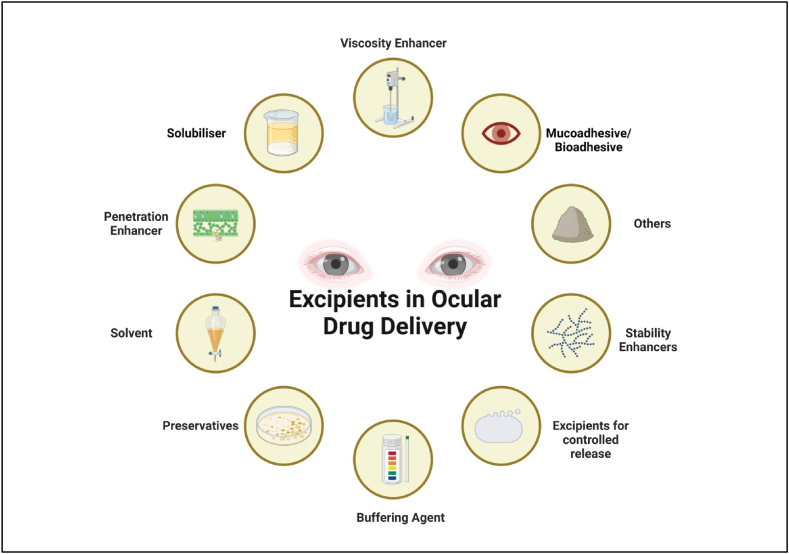
Excipients utilized in ophthalmic drug delivery systems (Created with BioRender.com).

Cyclodextrins have become the focus of various research studies for decades as substances capable of increasing drug solubility. Because of the development of inclusion complexes, they solubilize sparingly soluble actives [160]. Cyclodextrins are cyclic oligosaccharides that have been relatively studied for their skill to augment drug bioavailability, solvability, therapeutic activity, permeability, and physicochemical stability while reducing toxicity and tissue irritation. The primary mechanism by which cyclodextrins improve the effectiveness of ocular systems is through their skill to upsurge the dissolution of lipotropic drugs in tears. Additionally, β-cyclodextrin has been found to increase corneal perviousness by confiscating cholesterol from corneal cells, thus enhancing drug conveyance [161]. Researchers have explored various approaches to utilize cyclodextrins in ocular preparation conveyance. For example, the invention of a cyclodextrin-thiolated-poly-aspartic-acid couple for the ocular conveyance of prednisolone established protracted drug announcement equated to the physical amalgamation of polymer then cyclodextrin [162]. Sulphobuthyether-cyclodextrin (SBE-CD) has also been used to increase ocular drug residency and enhance endo-ocular bioavailability, as shown by the inclusion complex of SBE-CD with chloramphenicol [160]. Thiolated cyclodextrins have shown promise in enhancing drug delivery. For instance, α-cyclodextrin coupled with cysteamine (α-CD-Cys) demonstrated significantly higher mucoadhesive and improved coverage of irritations compared to α-cyclodextrin alone, enabling efficient delivery of the parsimoniously solvable drug cetirizine [163]. Another strategy involves incorporating cyclodextrins into nanosystems, as demonstrated by the preparation of nanoparticles treated with an immunopositive therapeutic for the managing of parched eye ailment, where the fraction of α-CD-cyclosporine A to γ-cyclodextrin was carefully modulated to act as an aggregating agent [164]. Table 4 describes excipients used in modified-release ocular formulations. Fig. 4 describes various excipients used for ophthalmic formulation development.

**Table 4:** Excipients utilized in modified release ocular formulations, along with their possible benefits and drawbacks.

Excipient	Properties	Benefits	Drawbacks	References
Poly(ethylene glycol) (PEG)	Increase viscosity	Biocompatible, soluble in water, and thermos-active	When compared with other synthetic polymers, this one degrades quickly. Unstable between 20 and 25 °C The measure obligatory to tempt gelation is substantial, which distresses osmolarity.	[99]
Poly(vinyl alcohol) (PVA)	Increase viscosity	The slow rate of degradation	strong solvents utilized in the synthesis	[165]
Poly(glycolic acid) (PGA)	Increase viscosity	fast rate of degradation	fragile mechanical characteristics	[166]
Poly(glycolic acid – co – lactic acid) (PLGA)	Increase viscosity	The most popular polymer is utilized in ocular medication delivery, with an adaptable rate of degradation and water solubility.	Byproducts of acidic degradation	[167]
Poly(lactic acid) (PLA)	Increase viscosity	Easy to process, synthesized from natural sources	The slow rate of degradation	[168]
Poly(orthoester) (POE)	Increase viscosity	surface erosion degradation	not extensively researched for medication delivery applications	[168]
Poly(methacrylates) and derivatives (PMMA)	Increase viscosity	a well-known ocular polymer that is affordable	Non-biodegradable	[122]
Poly(acrylic acid) PAA	Increase viscosity	mucoadhesive, highly easily soluble in water	breakdown into acidic byproducts	[126]
Gelatin	Increase viscosity	Gelling, emulsifying, plus foaming appearances fairly accessible Inexpensive Not antigenic like collagen Groups may be chemically changed	Not for long-tenure drug conveyance arrangements. The low percentage of dilapidation	[132]
Collagen	Increase viscosity	Biocompatible, biodegradable, bioactive, and has a long antiquity of practice in medicine	Perils of immunogenicity, varying quality, and animal source issues	[136]
Dextran	Increase viscosity	High biocompatibility	It is hard to functionalize.	[133]
Chitosan	Mucoadhesive	Biodegradable Biocompatible Antioxidant that is nontoxic and antimicrobial. Cancer cell proliferation is slowed.	End products with a high degree of variability	[4]
Hyaluronic acid	Mucoadhesive	Enhances tissue hydration and lubrication Enhances tissue resistance widely accessible Completely biocompatible and biodegradable Eliminates Free radicals.	Minor adverse effects	[146]
Carboxymethylcellulose	Mucoadhesive	biodegradable, pH-sensitive, biocompatible, and capable of providing extended release	It is difficult to create appropriate viscous solutions.	[96]
Guar Gum	Mucoadhesive	Biodegradable, Mucoadhesive, anti-inflammatory, inexpensive, stable, and biocompatible	Brittleness, rapid swelling, poor hydrogel mechanical characteristics, and low recovery	[23]
Pullulan	Mucoadhesive	It is easily produced, stable, has good film-forming capabilities, is biodegradable, and is non-toxic.	Extremely slow diffusion necessitates drug functionalization.	[158]

## 6 Limitations of various excipients

**Fig. 5:**
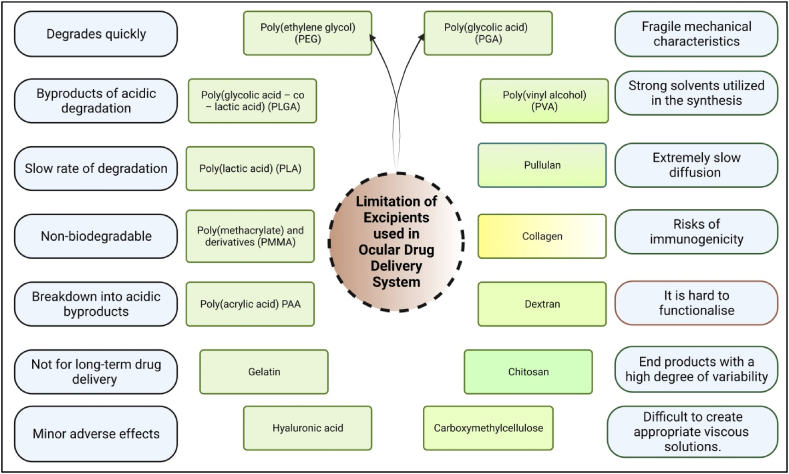
Summarization of various limitations/drawbacks of excipients utilized for ocular drug delivery (Created with BioRender.com).

The main obstacle in ocular drug delivery systems is attaining and maintaining an appropriate amount of drug in the bull's eye turf for a sufficient stretch period. So far, it is estimated that the topical route of administration could not achieve more than 5 % drug penetration in the eyeball. As a result, excipients play an essential role [169]. Excipients such as viscosity enhancers, chelating agents, and surfactants promote permeability; however, the dangers outweigh the advantages, resulting in eye discomfort, impaired vision, and altered stability. Additionally, additional preservatives impact safe medication consideration and medicine packaging compatibility [170]. Ocular excipients have various drawbacks, including low solubility, instability, or toxicity [171], which make them unsuitable for long-term safe use, universal adoption, or desired efficacy. Excipient constraints in ocular devices for drug delivery are addressed in Table 5. When utilized for an extended period, preservatives such as Benzalkonium chloride and Thiomersal can induce eye irritation, dry eye symptoms, and potentially harmful effects on the tear crust and corneal surface. The current trend of substituting Boric acid and Borax for the items mentioned above is unsafe, as it is not specified in the USFDA monograph for ophthalmic medicines. Furthermore, most excipients have solubility issues, and formulation variation has been recorded if the polymer is modified to improve release. The research is focusing on biopolymers; however, the supply is uncertain. Fig. 5 describes the several limitations of excipients used in ocular drug delivery.

**Table 5:** Overview of Limitations of various Excipients used in ocular drug delivery.

Name of Excipients	Role	Limitation due to	References
Benzalkonium chloride	Preservatives	Toxicity, incompatibility with anionic surfactant, hygroscopicity	[172]
Boric acid and borax	Preservatives	Not mentioned in the USFDA monograph for ophthalmic preparations.	[173]
Carbomer	Permeability enhancer polymer	Blurred vision	[174]
Carbopol	Permeability enhancer polymer	A sonication process is required	[175]
Carboxymethyl Cellulose (CMC)	Permeability enhancer polymer	Less corneal retention time	[176]
Chitosan	Permeability enhancer polymer	Final product variability	[177]
Gelatin	Permeability enhancer polymer	Rapid dissolution in aqueous environment	[178]
Guar gum	Permeability enhancer polymer	Limited solubility	[23]
Hyaluronic acid	Permeability enhancer polymer	Increase in intraocular pressure, rapid drug release in Intravitreal injection	[179]
Hydroxypropyl Methyl Cellulose (HPMC) nanoparticles	Permeability enhancer polymer	Slow drug release	[180]
Phenyl mercuric acetate	Preservative	Toxicity	[181]
Poloxamer	Permeability enhancer polymer	Wobbly on otherwise after 20–25 °C, quantity sought to accomplish gelation is high, distressing osmolarity	[182]
Poly Lactic co-glycolic acid (PLGA)	Permeability enhancer polymer	Delayed release	[23]
Polyglycolic acid (PGA)	Permeability enhancer polymer	Limited solubility	[23]
Thiomersal	Preservative	Toxicity	[183]

## 7 Toxicity studies of excipients

Toxicities primarily transpire in the fore slice of the eyes due to the high numbers of trial jots at the spot of topical direction. As a result, toxic effects are most frequently pragmatic in the conjunctiva, cornea, iris, surrounding fleshes and organs for illustration, eyelids, skin, and lacrimal glands. It is crucial to develop and deliver safe and well-tolerated dosage forms. Below are various tests to ensure the ocular excipients' safety [184].

### 7.1 MTT assay

The short-term exposure test method involves the quantitative measurement of cell viability by assessing the catalytic renovation of the vital tint MTT (3-(4,5-Dimethylthiazol-2-yl)-2,5-diphenyltetrazolium bromide), also acknowledged as Thiazolyl Blue Tetrazolium Bromide, which results in the production of a blue formazan salt by living cells. This measurement is conducted after extracting the cells. The resulting cell viability, obtained after a 5-min acquaintance, is equated to a solvent control (relative viability) and is cast-off to judge the latent eye peril of the trial chemical conferring to the OECD guidelines [185].

### 7.2 Draize eye trial

Albino rabbits, particularly New Zealand whites, are often used for ocular testing. Before exposure, the animals must be acclimated to the testing environment. It is also essential to assess the animals beforehand to ensure they have normal eyes, and the cages should be designed to minimize the risk of accidental injury. Typically, 3 to 6 creatures are cast off for each trial prescription, and in some cases, a solo creature may oblige as a sentry beforehand for further acquaintances [185,186]. Supplementary trial creatures may be counted to guesstimate the upshot of the vehicle if it is not hitherto acknowledged. During the experiment, one eye is assigned as the experimental eye, while the opposite eye is a matched control and is generally untreated. The experimental substance is administered in a volume of 0.1 ml per eye, although smaller doses (e.g., 0.01 ml) or single eye drops (approximately 0.04 ml) may be additional suitable. Solid amalgams are typically pulverized into fine dust before application. The experimental substance is applied to the lower conjunctival fornix, allowing natural blinking. Sometimes, the eyelids can be gently closed for a few seconds after application [185,186]. If needed, hygienic saline or a sensible brackish solution can be operated to flush the ocular outward. Pre- and post-exposure assessments are conducted through external visual examination under suitable lighting conditions [185,186]. Additional information may be gathered through magnification using binocular loupes or slit-lamp biomicroscopy. Evaluations are typically carried out at specific time intervals, such as 1, 24, 48, and 72 h after exposure, and if necessary, at 7 and 21 days. Ocular changes are evaluated using a scoring system that considers any eyelids, conjunctiva, cornea, and iris modifications. Supplementary tests, such as measuring levels of the test substance in tissues and fluids, may also be performed. Histopathological examination of removed eyes is generally reserved for severe reactions [[185], [186], [187]].

### 7.3 Isolated/enucleated organ/organotypic methods

Optometric and spectroscopic methods are commonly employed in protocols for evaluating ocular effects to quantitatively assess changes in the isolated cornea caused by a test substance. These methods are usually followed by histological analysis to provide further insights. Corneal imperviousness is an important *In vivo* terminus for assessing the things on the cornea, although the data obtained is often based on observational and subjective assessments. Corneal opacity indicates various changes in the cornea, such as protein denaturation, bump, and vacuolization, then hurt to the epithelium, and corneal stroma [188].

### 7.4 HET CAM test

To prepare the eggs for testing, fresh, fertile eggs undergo a cleaning process using 70 % alcohol. The eggs are nurtured at a controlled temp. of 37 ± 0.5 °C plus a relative humidity of 66.0 ± 5.0 % for a duration of 8 diurnal. During the nurture period, the eggs are physically rotated every 720 min around the equatorial axis to thwart the embryo from sticking to the shell. On the one-eighth diurnal, the eggs are taken out of the incubator and inspected using a flashlight to confirm the presence of embryo development. Careful incisions are made in the eggshells to expose the Chorioallantoic Membrane (CAM) surface to create windows. Positive control solutions such as 0.5 M NaOH are used to assess coagulation, acetone is used to evaluate hemorrhage, and propylene glycol (PG) is used to observe hyperemia. A negative control solution, phosphate buffer solution (PBS) per a pH of 7.4, is also employed. The testing solutions are applied to the clearest vessel of the CAM, and their effects are compared to the control solutions. After each sample application, the CAM morphology is evaluated for irritation reactions, including blood coagulation, bleeding, and hyperemia. These reactions are assessed at 30 s, 2 min, and 5 min of exposure, and the irritation potential of the sample is calculated based on these observations [189].

### 7.5 In silico methods

In silico mockups exploit storehouses of prevailing toxicology data derived from *Ex vivo* and I*n vivo* tryouts to forecast the venomousness of samples. These models rely on quantitative structure-activity relationships (QSAR) to launch the linkage mid a trial's chemical assembly and biological possessions. QSAR is rooted in the principle that the bustle of jots can be anticipated based on their assembly, plus these projections can be quantified [190]. By incorporating biological responses into computational algorithms, predictive models can be generated. Developing a dependable QSAR model necessitates accumulating, assessing, and weighing a sufficient volume of statistics for a definite noxious logic terminus [190]. This process facilitates a comprehensive connection between the jots under scrutiny and their biological bustle. Excipients augment drug conveyance by taming solvability, governing pharmacokinetics, and prolonging announcement. However, in ocular delivery, safety is of utmost concern [190,191]. Furthermore, Table 6 describes the list of excipients along with their toxicity studies.

**Table 6:** These characteristics help certify the well-being and use of ocular preparation conveyance arrangements.

Name of Excipients	Type of Toxicity studies performed	Outcome	References
Benzalkonium chloride	MTT Test	Not safe	[192,193]
Benzyl alcohol 4 %	Rabbit ST injection	Not Safe	[194]
Carboxymethyl cellulose 1 %	Rabbit sub-Tenon injection	Not safe	[194]
Chitosan nano particles	Draize Test	Safe	[195]
EDTA	MTT Test	Not safe at higher concentration	[192]
Hydroxy ethyl cellulose	Draize Test, MTT Test	Safe	[196]
Hydroxy propyl methyl cellulose	Draize Test, MTT Test	Safe	[196]
Macrogolglycerol hydroxy stearate (MGHS40)	MTT Test	Not safe	[197]
Methylcellulose 0.25 %	Rabbit ST injection	Safe	[194]
Poloxamer 15.3 %	HET CAM, MTT Test, Draize ocular	Safe	[189]
Polyethylene glycol	ST injection in rabbits	Safe	[194]
Poly Lactic co-glycolic acid (PLGA): Forskolin (F) than rutin (R)-encumbered chitosan (CS)-coated poly lactic-*co*-glycolic acid (PLGA) nanoparticles (NP)	HET CAM test	Safe	[198]
Polyvinyl alcohol (PVA)	MTT Test	Safe	[189]
Polyvinylpyrrolidone (PVP)	Draize Test, MTT Test	Safe	[196]
Thiolated Cyclodextrin	Draize ocular	Safe	[199]
Transcutol 1.33 %	HET CAM, MTT Test, Draize ocular	Safe	[189]
Triacetin 5 %	HET CAM, MTT Test, Draize ocular	Safe	[189]

## 8 Future directions

Ocular drug delivery is a complex process, making ensuring patient compliance and user-friendliness difficult. However, there is significant potential for improvement through research and development. Intelligent drug delivery systems offer promising solutions, including intravitreal injections, in situ gels, nanoparticles, implants, eye devices, and coils [59]. Another area of emphasis is the creation of processing excipients that enhance medication stability. Ocular medications frequently suffer degradation issues in the ocular environment. Future research can focus on developing excipients that preserve sensitive medications against degradation, allowing them to maintain therapeutic efficacy over long periods [169]. This could entail the introduction of innovative polymers or additives to form a protective barrier around the medication molecule, sheltering it from environmental conditions and extending its shelf life. Another crucial element of ocular medication administration is increasing drug solubility [18]. Many medicines used in ophthalmic therapy are poorly soluble, limiting their efficient distribution to target tissues. Processed excipients can be adjusted to improve drug solubility, allowing for more efficient and effective ocular delivery. Advanced formulation approaches, i.e., the creation of self-emulsifying systems or lipid-based carriers, could be investigated to improve drug solubilization and absorption inside the ocular tissues [169]. Adding permeation enhancers or mucoadhesive excipients into ocular formulations may enhance drug capture and retention, allowing improved penetration into ocular tissues. Nanotechnology has enormous promise for ocular preparatory conveyance in processed excipients. Nanoscale excipients can be designed to provide besieged and continuous drug announcements, allowing for more significant positive results [18]. Furthermore, processed excipients with improved healing, such as in situ gels, implants, or contact lenses, can revolutionize ocular treatment delivery. These arrangements can be planned to bring together processed excipients, allowing for sustained medication release, extended residence time, and increased patient compliance. Continued research and development in this subject will result in the creation of personalized excipients that optimize drug distribution, improve therapy effects, and rally for the overall handling of ocular illnesses [18,169,200,201].

## 9 Conclusion

Ocular preparatory transmission is a complex and continuing experience that necessitates the development of effective and targeted strategies. Although several methods are available, such as topical drops, injections, and systemic delivery, sustaining effective medication concentration for a prolonged time creates challenges. Significant advances in materials engineering and pharmacological delivery strategies are being pursued to address these constraints. Innovative drug conveyance arrangements intended for ocular preparation conveyance have emerged as viable options, intending to increase bioavailability, improve treatment effects, and overcome obstacles associated with conventional therapy. Nanoparticles, hydrogels, microparticles, and implantable devices are examples of materials that provide prolonged release, controlled delivery, and targeted dispersion within ocular tissues. As a vital part of ocular formulations, excipients influence drug conveyance qualities. Researchers are looking for new excipients that are changing current ones to improve medication release, solubility, bioavailability, and residence time inside ocular tissues. To guarantee patient safety and limit unwanted effects, these excipients' safety profiles and possible toxicology must be thoroughly investigated. Despite tremendous advances, problems such as enhancing eye penetration, effective drug release kinetics, biocompatibility, and system stability persist. The combination of nanotechnology and targeted drug delivery systems shows promise, taking advantage of the distinctive characteristics of nanoparticles for precise and streamlined drug release. The future of ocular medication administration depends on the continuing research of innovative methods, excipient optimization, and nanotechnology breakthroughs. Challenges associated with ocular preparation conveyance can be addressed through cross-disciplinary collaboration and thorough research, increasing handling outcomes for affected roles with ocular diseases.

## Funding

Not Available.

## Data availability statement

There is no research related data stored in publicly available repositories and the data will be made available upon request.

## CRediT authorship contribution statement

**Sumel Ashique:** Writing – review & editing, Funding acquisition, Formal analysis, Data curation, Conceptualization. **Neeraj Mishra:** Validation, Methodology, Investigation, Funding acquisition. **Sourav Mohanto:** Writing – review & editing, Visualization. **B.H. Jaswanth Gowda:** Funding acquisition, Formal analysis, Data curation. **Shubneesh Kumar:** Visualization, Validation, Resources. **Amisha S. Raikar:** Project administration, Methodology, Formal analysis, Conceptualization. **Priya Masand:** Formal analysis, Data curation. **Ashish Garg:** Visualization, Validation, Resources, Project administration. **Priyanka Goswami:** Visualization, Validation, Formal analysis. **Ivan Kahwa:** Writing – review & editing, Writing – original draft, Supervision, Resources, Data curation, Conceptualization.

## Declaration of competing interest

The authors declare that they have no known competing financial interests or personal relationships that could have appeared to influence the work reported in this paper.
